# Acute ST Segment Elevation Myocardial Infarction and Massive Pericardial Effusion Due to Infective Endocarditis

**DOI:** 10.5811/cpcem.2016.12.32674

**Published:** 2017-03-16

**Authors:** Maxwell Thompson, David C. Pigott, John Gullett, Blayke Gibson

**Affiliations:** The University of Alabama at Birmingham, Department of Emergency Medicine, Birmingham, Alabama

## Abstract

Chest pain is a common complaint evaluated in the emergency department. While chest pain in a 22-year-old patient is typically a complaint of low acuity, high-acuity cases that rival those of the older patient population are well documented. We describe a case of complicated infective endocarditis in which point-of-care ultrasound (POCUS) aided the diagnosis of ST-elevation myocardial infarction secondary to a septic thrombus in a 22-year-old female with a history of intravenous drug use. Emergency physicians should be aware of the rare high-acuity cases as well as the impact of POCUS on rapid clinical assessment and treatment of patients of all ages presenting with chest pain.

## INTRODUCTION

Chest pain, one of the most common emergency department (ED) complaints, is associated with numerous underlying etiologies with varying treatment and management strategies. Chest pain with accompanying ST segment elevation often places a patient in a pre-defined, institution-dependent pathway involving an interventional cardiologist and/or intravenous thrombolysis due to a presumed atherothrombotic event. In a young patient without significant risk factors for coronary artery disease, it is important to remember that, although rare, other life-threatening causes of ST segment elevation myocardial infarction (STEMI) exist that may require specific management strategies.

## CASE REPORT

**Image 1:**
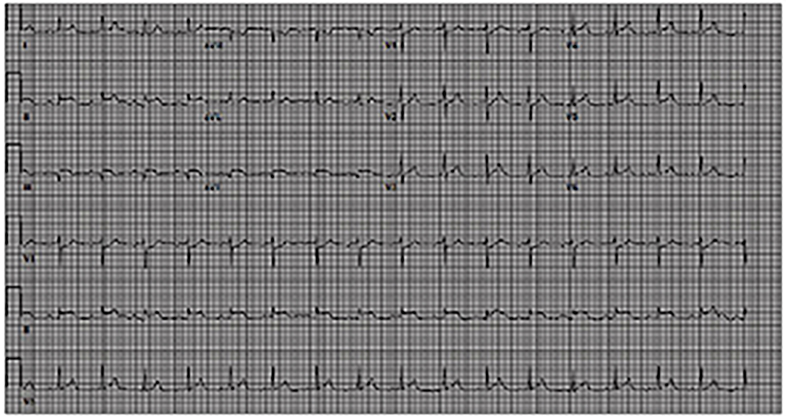
Electrocardiogram revealing sinus tachycardia with ST segment elevation in leads II, III, aVF.

A 22-year-old white female with a history of intravenous drug use (IVDU) presented to our ED with a two-day history of sharp, substernal chest pain. She had been in a substance abuse rehabilitation facility when her chest pain became so severe that emergency medical services was called. On arrival to the ED, an electrocardiogram revealed sinus tachycardia with STEMI in a right coronary artery (RCA) distribution (Image 1). Cardiology was consulted and the patient was prepped for percutaneous intervention for revascularization. Given the patient’s age, history of IVDU and lack of risk factors for primary cardiac disease, point-of-care ultrasonography (POCUS) was performed by emergency physicians to evaluate for other causes of the patient’s presentation.

**Image 2:**
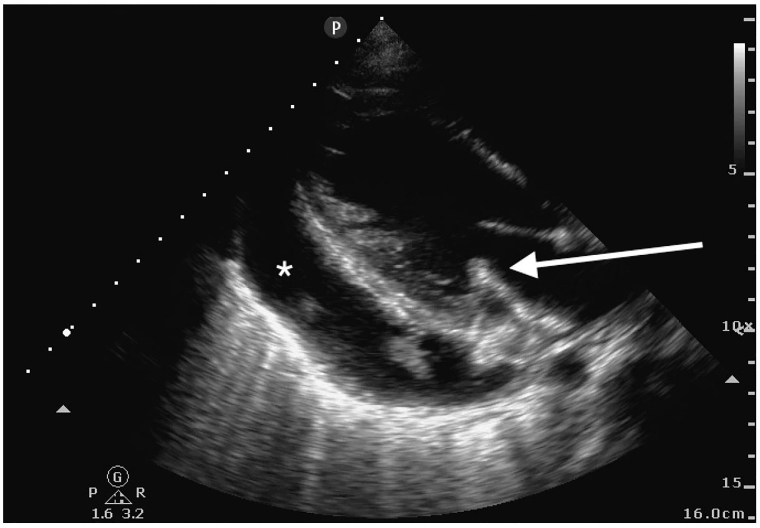
Parasternal long-axis transthoracic view showing pericardial effusion (star) and thickened posterior mitral valve leaflet (arrow).

POCUS revealed a large pericardial effusion with fibrinous stranding without evidence of tamponade physiology (Image 2). Additionally, a thickened posterior mitral valve leaflet was noted, concerning for vegetation (Image 2). The remainder of the cardiac exam, aortic root and thoracic aorta appeared normal in appearance.

**Image 3:**
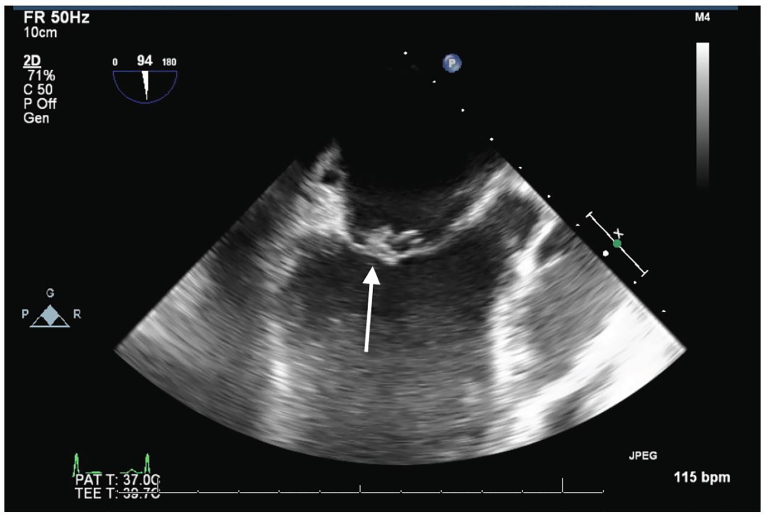
Transesophageal echocardiography Midesophageal longitudinal image of mitral valve showing vegetation (arrow) on anterior leaflet.

The patient was taken emergently for revascularization. Left heart catheterization (LHC) showed a large infectious thrombus causing 100% occlusion of the mid-RCA. This thrombus was aspirated and coronary flow was reestablished. Due to the risk of infection, stenting was not performed at that time. Transesophageal echocardiography (TEE) showed large vegetations on the anterior and posterior mitral valve leaflets with severe mitral regurgitation (Image 3). The patient became acutely hypotensive during the procedure and an emergent intra-aortic balloon pump was placed for hemodynamic support.

The patient was transported to the cardiac intensive care unit in critical condition. She subsequently developed worsening cardiogenic shock and expired shortly after admission.

## DISCUSSION

This patient’s diagnostic evaluation was complicated by an atypical presentation of infective endocarditis (IE). The patient presented to the ED with chest pain associated with ST segment elevation and a recent history of fever and chills in the setting of known IV polysubstance abuse. IE is a not uncommon complication of IV drug abuse with incidence ranging from 1.7 to 6.2 cases per 100,000 person-years.^1^

Several factors make IV drug users susceptible to IE. First, venipuncture using nonsterile technique can lead to bacteremia. Secondly, the caustic effect of both the drug injected as well any adulterants (e.g., talcum powder) may cause subclinical damage to the myocardial endothelium, predisposing the surface for vegetation accumulation.^2^

Additionally, underlying structural lesions in the endocardium can predispose a patient to developing left-sided bacterial endocarditis. Mitral valve prolapse is implicated in a large number of such cases in the industrialized world, while rheumatic heart disease commonly affects those in developing countries.^1^,^3^,^4^

IE is most often due to a single organism with *Staphylococcus aureus* being the most implicated organism. Other organisms commonly seen include streptococcal species, Pseudomonas, and enterococci. Polymycrobial endocarditis is also seen with IVDU and carries a high morbidity and mortality.^5^

Sequelae of left-sided endocarditis are most often due to thromboembolic events that occur when infected materials are dislodged from the endocardium and enter the systemic circulation. Septic emboli may affect virtually any organ system. Janeway lesions, splinter hemorrhages, and more serious complications such as stroke, renal and splenic infarction are well described.^6^ In our case, the patient also sustained multiple splenic infarctions diagnosed on chest computed tomography (CT) angiogram, as well as the septic coronary artery embolism causing the patient’s acute MI.

There are also a number of immunologic features of IE and Osler’s nodes. Immunologic factors are also implicated in the development of pericardial effusions.^7^,^8^

The etiology of pericardial effusions in patients with mitral valve IE is likely multifactorial. As previously discussed, the proinflammatory state and high titers of circulating immune complexes can certainly play a role. Additionally, a periannular abscess can lead to development of an aortocavitary fistula, causing severe pericardial effusions and poor outcomes.^8^

## CONCLUSION

The case presented here is unique in the fact that the mitral valve vegetation was readily identifiable on point-of-care ultrasound by emergency physicians. With the history of fevers, IVDU, splenic infarcts on CT and the vegetation seen on ultrasound, it was clear that the cause of this patient’s STEMI was likely due to infective endocarditis, rather than atherosclerotic coronary artery disease. Despite the patient’s poor outcome, this case demonstrates the utility of point-of-care ultrasound in the diagnosis of specific cardiac abnormalities that led to a rapid diagnosis of the patient’s underlying disease, and the initiation of potentially life-saving interventions.

## 

Video 1 Parasternal long-axis transthoracic view showing fibrinous pericardial effusion and thickened posterior mitral valve leaflet.
